# Association between Days Open and Parity, Calving Season or Milk Spectral Data

**DOI:** 10.3390/ani13030509

**Published:** 2023-02-01

**Authors:** Liangkang Nan, Chao Du, Yikai Fan, Wenju Liu, Xuelu Luo, Haitong Wang, Lei Ding, Yi Zhang, Chu Chu, Chunfang Li, Xiaoli Ren, Hao Yu, Shiyu Lu, Shujun Zhang

**Affiliations:** 1 Key Laboratory of Agricultural Animal Genetics, Breeding and Reproduction of Ministry of Education, College of Animal Science and Technology, Huazhong Agricultural University, Wuhan 430070, China; 2 Henan Institute of Science and Technology, College of Animal Science and Veterinary Medicine, Xinxiang 453003, China; 3 Hebei Livestock Breeding Station, Shijiazhuang 050000, China

**Keywords:** days open, parity, calving season, mid-infrared spectra

## Abstract

**Simple Summary:**

The days open was calculated as the difference between the dates of successful insemination and previous calving, which is a critical trait for the dairy industry. However, factors affecting days open were not well documented, and clarifying the mechanism of days open will contribute to the high-quality development of the dairy industry. Therefore, the objectives of this study are to evaluate the difference in days open between parity or calving season groups and assess associations of days open with wavenumbers ranging within the mid-infrared region. We found that the days open in calving season groups were significantly different, and dairy cows having a calving event in autumn showed the shortest number of days open. Weak associations were detected between days open and wavenumbers spanning the mid-infrared region. Based on the findings of the study, the reproduction management of dairy herds can be dynamically optimized according to the calving seasons, and the relationship between wavenumbers and days open needs to be further confirmed to service the dairy industry better.

**Abstract:**

Milk spectral data on 2118 cows from nine herds located in northern China were used to access the association of days open (DO). Meanwhile, the parity and calving season of dairy cows were also studied to characterize the difference in DO between groups of these two cow-level factors. The result of the linear mixed-effects model revealed that no significant differences were observed between the parity groups. However, a significant difference in DO exists between calving season groups. The interaction between parity and calving season presented that primiparous cows always exhibit lower DO among all calving season groups, and the variation in DO among parity groups was especially clearer in winter. Survival analysis revealed that the difference in DO between calving season groups might be caused by the different P/AI at the first TAI. In addition, the summer group had a higher chance of conception in the subsequent services than other groups, implying that the micro-environment featured by season played a critical role in P/AI. A weak linkage between DO and wavenumbers ranging in the mid-infrared region was detected. In summary, our study revealed that the calving season of dairy cows can be used to optimize the reproduction management. The potential application of mid-infrared spectroscopy in dairy cows needs to be further developed.

## 1. Introduction

The days open (DO) was calculated as the difference between the dates of successful insemination and previous calving, and it was a critical trait with a complex genetic nature that contributes a lot to the total milk yield over lactation and further to the profitability of the farms [1,2,3,4]. The estimated heritability of DO reported in previous studies ranges from 0.02 to 0.11 [4,5,6,7], indicating that environmental factors influence DO more than genetic factors do. Indeed, factors such as health status [8,9], estrus synchronization [10,11], heat stress [12], and body conditions [13,14] functioned indirectly on DO by their impacts on the chance to be conceived for dairy cows. Additionally, the relationship between DO and days dry [15] as well as services per conception [6] has been reported. Studies have also focused on the prediction of DO [16,17].

High-producing dairy cows are at high risk for negative energy balance (NEB) in early lactation [18]. Moreover, severe NEB is an important motivator of metabolic disorders [19,20] and compromised health and fertility [21,22]. Several studies have been shown that ovarian activity is related to energy status after calving [23,24]. To mitigate the metabolic pressure due to energy deficit, signaling pathways related to energy metabolism are activated, thereby inducing the changes in milk and plasma composition: for instance, fatty acid profiles, β-hydroxybutyrate (BHB), acetoacetate, acetone and so on [24,25,26]. The relationship between part of these molecules and DO has been reported by studies [4,27], and the contents of these components can be rapidly predicted from milk spectroscopy data [28,29]. Overall, the energy status, milk composition and fertility of dairy cows are interdependent.

Fourier-transform mid-infrared (FT-MIR) spectroscopy has been widely used to predict the contents of milk components [30]. The FT-MIR spectra consist of absorbances for 1060 wavenumbers across the range from 5010.15 to 925.66 cm^−1^ [31], corresponding to the short-wave infrared region (SWIR), mid-wave infrared region (MWIR), and long-wave infrared region (LWIR). The absorbance at each wavenumber resulted by the interactions between the infrared light and molecules is helpful to characterize the chemical composition in milk. In recent years, more studies have focused on the traits indirectly related to milk composition or complex traits of interest such as methane emissions [32,33,34,35,36,37], pregnancy status [38,39,40,41], and energy balance [42,43,44]. Recently, FT-MIR data have been innovatively used to identify the physicochemical characteristics [45] and genotype [46] of β-casein in milk.

As mentioned earlier, the DO of dairy cows is affected simultaneously by multiple factors, but not all of them have been well stated. Therefore, the objectives of this study are to evaluate the difference in DO between parity or calving season groups, assess associations of DO with each of the wavenumbers, and confirm the marginal and joint posterior probability of associations (PPA) of wavenumbers with DO.

## 2. Materials and Methods

### 2.1. Data Acquisition and Editing

A total of 5000 milk samples from 2456 non-pregnant Holstein dairy cows were collected between November 2019 and December 2021 from 9 herds located in Hebei Province, China. The milk samples were analyzed by an infrared spectrometer (Foss Electric, Hillerød, Denmark) to obtain the spectra data. Overall, 1060 transmittance values of the spectra data were transformed into absorbance using the equation: absorbance = log_10_ (1/transmittance). Then, the absorbance values were normalized to a null mean and a unit variance.

We calculated the standardized Mahalanobis distances for all spectra and preserved the spectra whose standardized Mahalanobis distances were not greater than three [38,47]. Milk fat and protein contents, which were predicted from the FT-MIR spectra, were also checked to be in the range of 1.5–9% and 1–7%, respectively [30]. Afterwards, the DO, calculated as the number of days between being conceived and previous calving, was required to be in the range of 25 to 400 d. To evaluate the fertility of dairy cows at early stages of lactation, only milk samples collected between 5 and 120 d of each lactation were retained. Finally, the total number of records used in this study was 3771 on 2118 cows from 9 herds. Table 1 shows the descriptive statistics for milk components and DO.

### 2.2. Difference in DO between Parity or Calving Season Groups

We performed a linear mixed-effects model and survival analysis to access the association between DO and parity or calving season. Survival analysis was performed on the records using the Kaplan–Meier estimator provided by the survival package in R [48]. A Mantel–Haenszel test was conducted to evaluate the difference between levels of factors [49]. The linear mixed-effects model was defined as:

DO_ijkl_ = μ + Parity_i_ + SeasonC_j_ + (Parity × SeasonC)_ij_ + Herd_k_ + ε_ijkl_.

where DO represents days open; μ is an intercept; Parity_i_ is the fixed effect of the ith parity (i = 1, 2, 3, ≥4); seasonC_j_ is the fixed effect of the jth calving season (spring, March–May; summer, June–August; fall, September–November; winter, December–February); Parity_i_ × seasonC_j_ is the interaction between the ith parity and jth calving season. Herd_k_ is the random effect of the kth herd (k = 1 to 9), and ε is the residual random error. The model was fitted using the lmer function provided by the lme4 package in R [50]. The effect was considered significant whenever the *p*-value related to the factor of interest was less than 0.05.

### 2.3. Associations between Days Open and Milk Spectral Data

**Figure 1:**
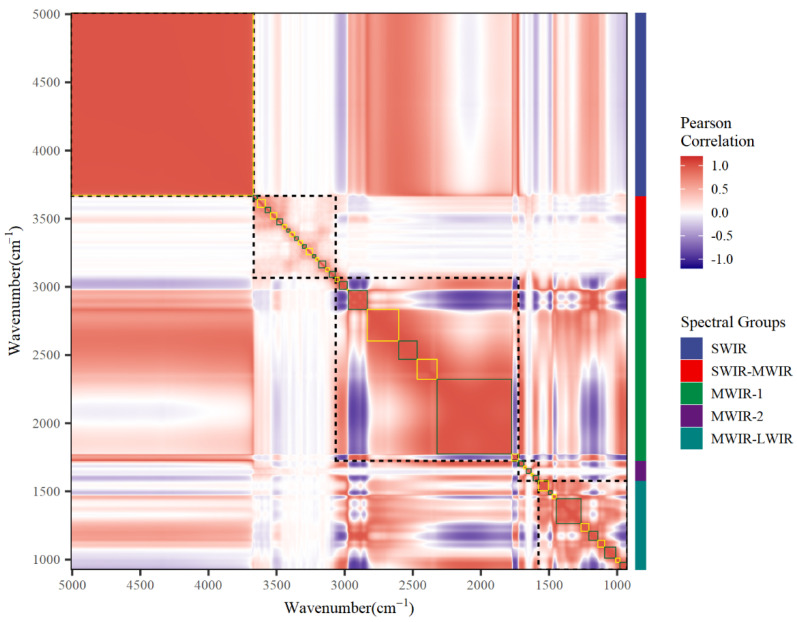
The correlation heatmap of wavenumber absorbances across 1060 wavenumbers ranging from 5010.15 to 925.66 cm^−1^. SWIR, short-wavenumber infrared or near-infrared; MWIR, mid-wavenumber infrared; LWIR, long-wavenumber infrared.

Considering the high correlation between absorption values of adjacent wavenumbers (Figure 1), associations of DO with milk spectral data were assessed jointly by the model:

DO = μ + X_w_β_w_ + X_f_β_f_ + X_r_β_r_ + ε

where DO is the days open, and μ is an intercept. X_w_ is the matrix representing all the 1060 wavenumbers, and β_w_ is the corresponding vector of effects, which is assigned to a mixture (known as BayesB) of a point of mass at zero and a scaled-t slab prior [51]. X_f_ is the matrix for the effects of parity, calving season, and DIM, and β_f_ is the corresponding vector of effects, which is assigned to a flat prior. X_r_ is the matrix for the effects of herds, and β_r_ is the corresponding vector of effects, which is assigned to a Gaussian prior. ε is the residual random error item. The treatments for parity and calving season here were the same as the linear mixed-effects model, and four DIM intervals were created in a monthly manner (5–30 d, 31–60 d, 61–90 d, 91–120 d).

The prior density of BayesB is characterized by 3 hyper-parameters: π, which is subjected to beta distribution, represents the probability of non-null effects. df*_β_*, which is a user-defined hyper-parameter, represents the degrees of freedom, and S*_β_*, which was subjected to Gamma distribution, indicates the scale of the scaled Student’s t-distribution. The extent of variable selection and shrinkage is controlled by these three hyper-parameters.

The BGLR function of the BGLR package in R was used to fit the data with the model defined above [52]. A total of 450,000 iterations of MCMC Gibbs sampling were performed with the first 100,000 iterations discarded as burn-in, and the remaining 350,000 circles were resampled with the thinning interval of 10 samples, thereby obtaining 35,000 samples for subsequent inference. Convergence of the models was inspected visually by trace plots.

### 2.4. Marginal and Joint Associations between Wavenumbers and DO

Under the variable selection and shrinkage mechanism of BayesB, the association of the wavenumbers of spectra with DO can be evaluated by the posterior probability of association (PPA) as previously reported by [29,40].

All 1060 wavenumbers in milk spectral data were clustered using an adjacency-constrained hierarchical agglomerative clustering algorithm (HAC). The adjclust package in R was used to implement HAC, and the optimal number of windows was determined by the slope heuristic method, as described by [53]. Both the marginal PPA and joint PPA were processed by min–max normalization to compare the relative importance among wavenumbers or clusters.

## 3. Results

### 3.1. Difference in DO between Parity or Calving Season Groups

**Figure 2:**
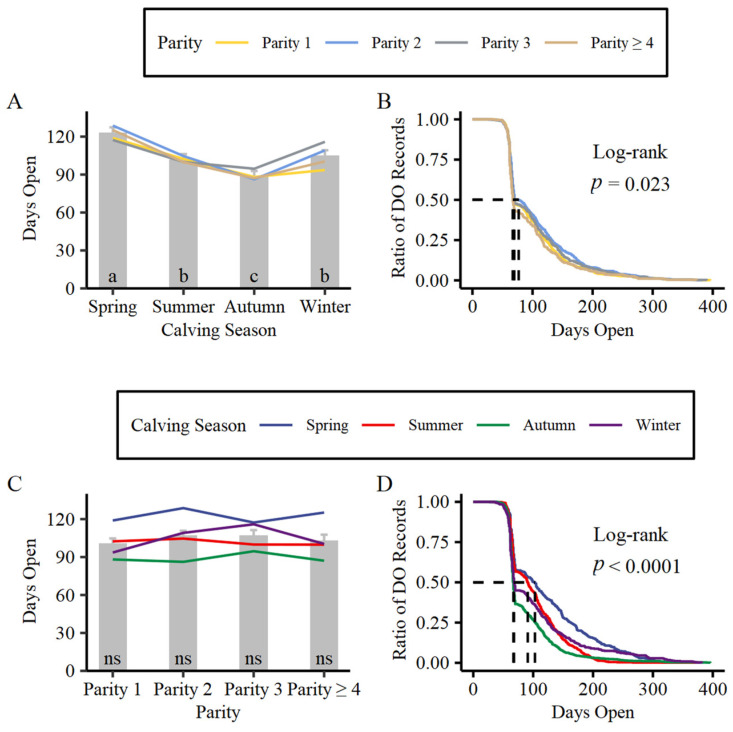
Least-squares means and corresponding standard error of DO at different levels of calving season (**A**) and parity (**C**) inferred by linear mixed model. Survival analysis of DO based on parity (**B**) and calving season (**D**) was carried out by the Kaplan–Meier method. The horizontal dotted line indicates the median survival rate in survival analysis. The vertical dotted lines indicate the median survival time at different levels of parity and calving season in survival analysis. Different letters show significant differences (*p* < 0.05)

The least-squares means (LSM) of DO for dairy cows whose parity equal 1, 2, 3, and ≥4 was 101 ± 3.72 d, 107 ± 3.79 d, 107 ± 4.36 d, and 103 ± 4.71 d (Figure 2C). No significant difference was observed between parity groups (*p* > 0.05). The DO of dairy cows whose calving seasons were spring, winter, summer and autumn showed a downtrend among parity groups except for primiparous cows (Figure 2C). Table 2 and Figure 2B demonstrated the survival analysis of DO based on parity. The median survival time in all parity groups was around 70 d (69.0 d, 77.0 d, 69.5 d, and 67.0 d for parity equals 1, 2, 3, and ≥ 4), and it was close to the lower confidence level.

The LSM of DO was 123 ± 4.34 d, 102 ± 4.18 d, 89 ± 3.84 d, 105 ± 4.25 d for dairy cows whose calving seasons are spring, summer, autumn, and winter, respectively (Figure 2A). DO of dairy cows was notably different between calving season groups (*p* < 0.05). The interaction between parity and calving season presented that primiparous cows always exhibit lower DO among all calving season groups, and the variation in DO among parity groups was especially clearer in winter (Figure 2A). Survival analysis (Figure 2D) revealed that the difference in LSM of DO among calving season groups might be caused by the different P/AI at the first TAI. To our knowledge, the first TAI in herds adopting synchronization strategy mainly occurred at 50~70 DIM, which is overlapped with the time window that showed a drastic drop in the survival curve. Additionally, the summer group had a higher chance of conception in the subsequent services than other groups, implying that the micro-environment molded by season played a critical role in the P/AI. The median survival time was 103 d, 91 d, 67 d, and 68 d for spring, summer, fall, and winter calving groups, respectively (Table 2).

### 3.2. Associations between Wavenumbers and DO

**Figure 3:**
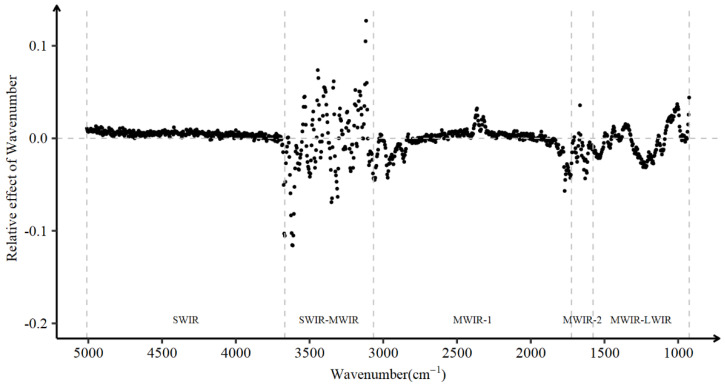
Relative effect of 1060 wavenumbers on DO. SWIR, short-wavenumber infrared or near-infrared; MWIR, mid-wavenumber infrared; LWIR, long-wavenumber infrared.

Consider the high correlation nature between absorption values of adjacent wavenumbers as shown in Figure 1, the relative effect of individual wavenumbers on DO was evaluated by an BayesB model (Figure 3). In general, negative effect values, which imply negative correlations between absorbance of wavenumbers and DO, were considered favorable for farms.

Wavenumbers in the SWIR region from 5010.15 to 3671.80 cm^−1^ had a relatively stable and near-zero relative effect on DO. This means that molecules in the milk whose chemical bonds interact with wavenumbers in this region were barely related to DO. The SWIR-MWIR region and the MWIR-2 region spanning from wavenumber 3667.94 to 3050.83 cm^−1^ and 1697.05 to 1585.20 cm^−1^, respectively, are characterized by the high variability of absorbances due to the absorption of water molecules. The effects of wavenumbers in the SWIR-MWIR region were evenly distributed around zero, but the effects of wavenumbers in the MWIR-2 region preferred to be slightly negative. A reasonable explanation for this phenomenon is that the high correlations between adjacent wavenumbers was extrapolated to the effects of wavenumbers on DO. Most wavenumbers located in the boundaries of the MWIR-1 region (wavenumbers from 3046.97 to 2641.99 cm^−1^ and 1978.60 to 1700.90 cm^−1^) had a negative effect on DO. The boundaries also contain absorption peaks for fat-B and fat-A, which range from 2873 to 2777 cm^−1^ and 1786 to 1725 cm^−1^, respectively [54,55,56]. Moreover, the absorbance of wavenumbers ranging from 2306.44 to 2387.44 cm^−1^ was positively associated with DO. The MWIR-LWIR region, known as the fingerprint region, is an important region for the prediction of milk components [57]. Wavenumbers from 1546 to 1492 cm^−1^ represent the absorption peaks of proteins in milk [54,56], and these wavenumbers had a negative effect on DO. Three wavenumbers at 1461.77 cm^−1^, 1238.07 cm^−1^ and 1110.79 cm^−1^ in this region exhibited a negative local minimum effect on DO, and these three wavenumbers have been reported to be related to the absorption of urea (1469 cm^−1^), fat (1460 cm^−1^), acetone (1238 cm^−1^), and lactose (1040 cm^−1^), respectively [54,56].

### 3.3. Marginal and Joint Associations between Wavenumbers and DO

The posterior probability of association (PPA) was also used to evaluate the associations between wavenumbers and DO. The marginal PPA of 1060 wavenumbers, as well as the joint PPA of windows clustered with highly correlated wavenumbers, were inferred by the BayesB model.

A total of 42 wavenumber clusters were identified by the HAC algorithm, and the number of wavenumbers per cluster ranged from 2 to 348 (Table 3). Since the windows were grouped based on the similarity of neighboring wavenumbers, we also visualized all the windows on the correlation heatmap using colored solid-line rectangles, as shown in Figure 1. The five spectral regions labeled SWIR, SWIR-MWIR, MWIR-1, MWIR-2, and MWIR-LWIR contain 1, 17, 9, 5, and 10 windows, respectively. In my view, the number of windows included in regions other than SWIR-MWIR and MWIR-2 can be considered as an indicator of their information volume. It is well documented that these two spectral regions are characterized by high variability in the absorbance and are responsible for the absorption of water molecules.

**Figure 4:**
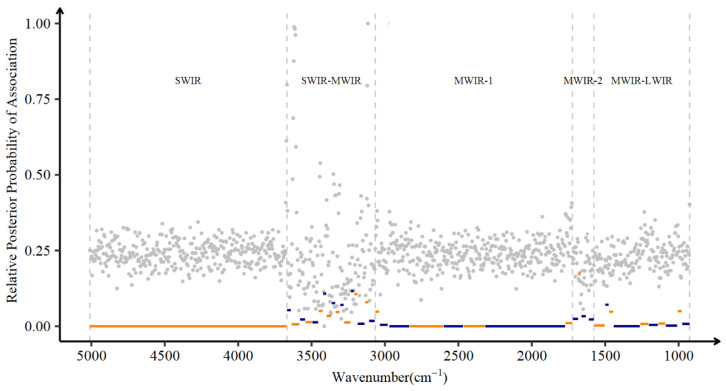
Relative posterior probability of association (PPA) of individual wavenumbers (gray dots) and windows clustered with wavenumbers (colored lines). SWIR, short-wavenumber infrared or near-infrared; MWIR, mid-wavenumber infrared; LWIR, long-wavenumber infrared.

Figure 4 shows the marginal PPA of individual wavenumbers as well as the joint PPA of clustered windows. Most marginal PPA fluctuated around 0.25, indicating a weak association between DO and wavenumber. Nevertheless, the wavenumbers with a larger relative effect on DO also show stronger marginal PPA with DO; examples include the wavenumbers located in the boundaries of the SWIR-1 region and the mentioned wavenumbers in the MWIR-LWIR region. In addition, except for the SWIR-MWIR and MWIR-2 regions, windows with slightly positive joint PPA are also located within the boundaries of the SWIR-1 and MWIR-LWIR regions. In summary, the results of marginal PPA and joint PPA were in line with the relative effects of wavenumbers.

## 4. Discussion

### 4.1. The Limitations of FT-MIR When Considering Complex Traits

Since FT-MIR data have been successfully applied to predict detailed milk composition [29,58,59,60,61], a large number of studies have been dedicated to developing the potential capabilities of FT-MIR such as the prediction of dairy cow health status [62,63,64], dry matter intake [65,66,67], weight [68,69], and heat production [70]. FT-MIR data also can be used to increase GWAS power to map genes associated with traits studied [71,72,73]. Days open, as a critical fertility trait, was also studied to determine its association with signals from FT-MIR spectra. Although some of the wavenumbers exhibit considerable effects on DO, the relatively low variance explained by the BayesB models reduced the predictive ability of FT-MIR spectroscopy to DO [47]. A similar dilemma arises in our study, where no wavenumbers or clusters of wavenumbers were found to be particularly associated with DO. For one thing, various environmental factors dominated the phenotypic variance of traits of interest, making great challenges to the robustness of the prediction model based on milk FT-MIR spectroscopy, and for another, the complex traits are partly or totally indirect with the molecules in milk whose concentrations are typically low enough and even under the reference threshold of detection of 100 ppm [74]. This is undoubtedly another major obstacle to the generalization and application of milk FT-MIR spectroscopy.

Studies that have made some useful attempts to break these limitations have been reported. Ref. [38] adopted a differences spectra strategy to enhance the signal-to-noise ratio when predicting the pregnancy status of dairy cows. Ref. [75] analyzed the same milk sample twice and removed the wavenumbers whose absorbance changed notably between measures to improve the signal-to-noise ratio. Currently, artificial intelligence technologies, especially deep learning, are making breakthroughs in various fields. Ref. [76] showed that the average Gard-CAM weights based on deep learning models can be used to select signals with important information during the model development procedure. Overall, we should improve the signal-to-noise ratio to highlight the wavenumbers that are truly associated with the traits of interest, such as the days open.

### 4.2. Factors Affect Days Open and the Association of DO with Wavenumbers

We investigated the differences in DO between the parity and calving season groups, and the results suggest that significant differences exist between the calving season groups. Dairy cows that underwent calving events in the fall presented the shortest DO compared to other groups. This is line with previous studies by [77,78]. The former study reported that the DO values of spring, summer, and autumn were 5.93, −2.66, and −3.53, respectively, compared to winter. The latter study reported DO values of 115, 104, 99, and 104 for the spring, summer, autumn, and winter calving seasons, respectively. Our study also found that summer had a higher probability of conception in the subsequent services than other seasons, implying that the micro-environment molded by seasons played an important role in the P/AI of services.

To our knowledge, the study by [47] was the first one evaluating the association of days open with wavenumbers of milk spectral data. Different from the way they deal with DIM (divided into four stages as monthly intervals), we treated DIM as a fixed effect in the BayesB model by assigning a flat prior to it. We also take parity and calving season into consideration when developing the BayesB model. Although the models were defined differently, the wavenumbers identified to be associated with DO were located in the same region. Benefiting from partitioning data into four parts according to DIM, [47] found that the DIM period 31 to 60 d seems to be most informative about the fertility of the dairy cow and is directly related to the milk lactation curve and its energy requirements.

## 5. Conclusions

Overall, our results suggest that the calving season of dairy cows can be used as an assistant tool to help farms optimize their breeding manage for better profits. A number of wavenumbers were also identified to be associated with days open of dairy cows, such as the wavenumbers at the boundaries of the MWIR-1 region, which are responsible for the absorption peaks of Fat-A and Fat-B, respectively, and wavenumbers located in the MWIR-LWIR region, which are related to the absorption of urea (1469 cm^−1^), fat (1460 cm^−1^), acetone (1238 cm^−1^), and lactose (1040 cm^−1^). These milk components could be regarded as effective indicators of the effects of animal metabolic and physiological status on fertility. However, the relatively low impact of these wavenumbers on days open suggests that predicting days open using FT-MIR only will be challenging.

## Figures and Tables

**Table 1:** Descriptive statistics of milk yield, milk components, and days open (DO).

Items	Samples	Mean	SD	CV	Min	Max
Yield, kg/d	3712	40.3	10.9	27.11%	5.2	79.3
Fat, %	3764	3.94	0.84	21.20%	1.50	7.83
Protein, %	3769	3.18	0.38	11.92%	2.06	5.31
Lactose, %	3769	5.23	0.23	4.38%	4.08	5.90
TS, %	3766	13.09	1.07	8.21%	9.74	17.56
SCS, log_2_(SCC/100) + 3	3767	2.14	1.67	77.85%	−3.64	6.32
Urea, mg/100 g	3765	13.2	3.5	26.60%	2.9	46.8
SnF, %	2743	9.04	0.43	4.74%	7.02	11.00
DO, d	3771	105.1	57.2	54.41%	27	396

**Table 2:** Summaries of survival analysis of DO based on parity and calving season.

Items	Events	Median	0.95 LCL	0.95 UCL
Parity				
1	869	69.0	68	76
2	726	77.0	69	88
3	380	69.5	67	89
≥4	282	67.0	66	70
Calving Season				
Spring	439	103	88	110
Summer	593	91	84	97
Autumn	767	67	66	67
Winter	458	68	67	70

**Table 3:** Lower and upper wavenumber limits of 42 windows (numbered from 1 to 42) adaptively clustered with 1060 wavenumbers.

Window	Lower (cm^−1^)	Upper (cm^−1^)	NW	Window	Lower (cm^−1^)	Upper (cm^−1^)	NW
1	925.66	975.80	14	22	2973.69	2977.55	2
2	979.66	1006.66	8	23	2981.40	3035.40	15
3	1010.51	1087.65	21	24	3039.26	3066.26	8
4	1091.51	1137.79	13	25	3070.11	3108.68	11
5	1141.65	1203.36	17	26	3112.54	3135.68	7
6	1207.22	1261.21	15	27	3139.54	3185.82	13
7	1265.07	1442.49	47	28	3189.68	3208.96	6
8	1446.35	1473.34	8	29	3212.82	3232.10	6
9	1477.20	1500.34	7	30	3235.96	3278.39	12
10	1504.20	1573.62	19	31	3282.24	3305.39	7
11	1577.48	1612.19	10	32	3309.24	3336.24	8
12	1616.05	1627.62	4	33	3340.10	3363.24	7
13	1631.48	1662.33	9	34	3367.10	3397.95	9
14	1666.19	1681.62	5	35	3401.81	3421.09	6
15	1685.48	1720.19	10	36	3424.95	3451.95	8
16	1724.04	1770.33	13	37	3455.81	3494.38	11
17	1774.18	2318.01	142	38	3498.23	3540.66	12
18	2321.87	2464.58	38	39	3544.52	3579.23	10
19	2468.43	2599.57	35	40	3583.09	3637.08	15
20	2603.43	2830.98	60	41	3640.94	3667.94	8
21	2834.84	2969.83	36	42	3671.79	5010.15	348

NW, number of wavenumbers.

## Data Availability

The data that support the findings of this study are available from the corresponding author upon reasonable request.
